# Loneliness enhances brand love for individualistic (but not collectivistic) consumers

**DOI:** 10.3389/fpsyg.2025.1586472

**Published:** 2025-10-03

**Authors:** Andy H. Ng

**Affiliations:** Cardiff Business School, Cardiff University, Cardiff, United Kingdom

**Keywords:** loneliness, brand love, cross-cultural differences, individualism, collectivism

## Abstract

**Introduction:**

It is not uncommon for people to experience loneliness. When people feel lonely, they are motivated to reestablish connections with other people directly or restore a sense of social connection indirectly through other means. As a brand symbolically connects all people affiliated with the same brand, constituting a broad social group, loneliness may motivate people to enhance their emotional attachment to a brand (i.e., brand love) to restore a sense of social connection indirectly through the brand. In the current research, I adopt a cultural lens to examine this proposition.

**Methods:**

Across two studies (Study 1: *N* = 200; Study 2: *N* = 267), I examined the moderating effect of culture on the causal effect of loneliness on brand love. Loneliness was manipulated using a recall task, and culture was measured and operationalized as individual differences in cultural orientation (Study 1) and racial background (Study 2).

**Results:**

For both studies, results indicated that culture moderated the effect of loneliness on brand love, such that loneliness caused an increase in brand love for individualistic consumers, but not collectivistic consumers.

**Discussion:**

These findings are consistent with current theorizing and empirical findings about cultural differences in how people conceptualize ingroup and relate to strangers who belong to the same broad social group.

## 1 Introduction

There has been a proliferation of research on brand love (see Gumparthi and Patra, 2020, for a review). The present research focuses on one factor that could potentially strengthen consumers’ love relationship with a brand; that is, consumer loneliness. However, I argue that whether loneliness would cause an increase in brand love depends on culture. Consistent to theorizing and empirical findings on differences in group conceptualization and processes between individualistic and collectivistic cultures (Yuki and Takemura, 2013), the present research shows that, for individualistic consumers, loneliness enhances their love towards a brand that they frequently use and are satisfied with its products. By contrast, for collectivistic consumers, loneliness does not have such a positive causal effect on brand love.

## 2 Loneliness and brand love

Humans generally enjoy the presence of close others and have a fundamental need for lasting and significant interpersonal relationships (Baumeister and Leary, 1995). This need to belong has an evolutionary basis in that it has survival and reproductive benefits (e.g., Barash, 1977; Buss, 1991). However, despite this fundamental motivation to establish and maintain social connections, individuals may feel that their current level of social connection does not meet their desired level. In this case, loneliness would arise (Perlman and Peplau, 1984; Weiss, 1973). Loneliness is not an uncommon experience. For example, according to a survey study on American adults conducted by Harris Poll, 72% reported having felt a sense of loneliness (Marcus, 2016). In England, according to the Community Life Survey, 21% of adult respondents reported that they never felt lonely (Department for Culture, Media and Sport, 2023). Loneliness is an aversive feeling, a form of social pain associated with the unmet need of belongingness that implicates the same neural region as does physical pain. Eisenberger et al. (2003) found that when participants were socially excluded (vs. not) in a virtual ball-tossing game, the anterior cingulate cortex (ACC) showed higher levels of activation, paralleling how the ACC is implicated in the affective component of physical pain (Rainville et al., 1997).

Although everyone is capable of feeling lonely, there are some known individual differences. For example, in a survey study with more than 40,000 participants across more than 200 countries, islands, and territories, it was found that men and younger people felt lonely more frequently (Barreto et al., 2021). Loneliness also differs as a function of socio-economic status. Wee et al. (2019) found that people living in a poorer physical environment reported higher levels of loneliness. In terms of personality correlates, all of the Big Five personality traits are associated with loneliness, such that loneliness is negatively correlated with extraversion, agreeableness, conscientiousness, and openness, and is positively correlated with neuroticism (Buecker et al., 2020). In addition, two of the Dark Triad traits—Machiavellianism and psychopathy—are positively correlated with loneliness in adolescents (Zhang et al., 2015). On the other hand, people who are high in trait empathy are less likely to experience loneliness (Beadle et al., 2012). With regard to consequences of loneliness, there is evidence that loneliness diminishes self-esteem (Cacioppo et al., 2006). According to sociometer theory (Leary and Baumeister, 2000), self-esteem (i.e., overall evaluation of the self) is a psychological system that monitors the degree to which one is socially valued. Thus, when people experience loneliness, they would perceive that they are not socially valued and their self-esteem would decrease as a result. Moreover, loneliness can have negative consequences for one’s physical health. For instance, loneliness is correlated with systolic blood pressure (Hawkley et al., 2006) and all-cause mortality (Shiovitz-Ezra and Ayalon, 2010). Furthermore, loneliness can have negative consequences for one’s cognitive abilities and mental health. For example, loneliness is associated with decline in general intelligence (Gow et al., 2007) and basic cognitive functioning (e.g., repeating a list of three unrelated objects, Tilvis et al., 2004) over time, as well as an increased risk for Alzheimer’s Disease (Wilson et al., 2007). Loneliness also longitudinally predicts symptoms of depression (but not the reverse) (Cacioppo et al., 2010).

When an individual experiences loneliness, this social pain serves as a warning signal that something is wrong (Jensen-Campbell and MacDonald, 2011), which triggers an approach motivation aiming at the repair and maintenance of social connections (Cacioppo et al., 2006). Indeed, loneliness increases people’s attention to social information and opportunities for social connections. For instance, individuals who are lonelier are more sensitive to social cues in faces and voices (Gardner et al., 2005). Likewise, the threat of social exclusion enhances people’s attention to smiling faces, which signal social acceptance (DeWall et al., 2009).

Loneliness also influences people’s behaviors in the consumption domain (see Huang and Li, 2023; Rawat et al., 2022; Shrum et al., 2023, for reviews). The loneliness-induced motivation to restore social connection gives rise to two broad consumption-based coping strategies - direct and indirect connection (see Shrum et al., 2023 for a review). For direct connection, people may engage in consumption activities that foster connection with other people directly. For example, social exclusion increases people’s tendency to buy a product that signals social group membership, spend money on an unappealing food product that is liked by an interaction partner, and even try an illegal drug if it increases social acceptance (Mead et al., 2011). Likewise, in a public context (i.e., when product preference would be known to others), consumers who are lonelier exhibit higher preference towards majority-endorsed products because they are more concerned about negative evaluation form others (Wang et al., 2012). Furthermore, lonely (vs. non-lonely) single people are more likely to engage in conspicuous consumption (i.e., buying and displaying expensive consumer products), driven by their increased desire for a romantic relationship (Liu et al., 2020). Finally, it has been found that loneliness increases people’s tendency to shop at physical retailers for the social experience (Rippé et al., 2018).

In addition to engaging in consumer behaviors that facilitate direct connection with others, lonely people may also turn to other consumption behaviors to restore a sense of social connection indirectly. To this end, people may relate to products or brands as surrogates for social connection, or use products because of their positive social primes. Loneliness is associated with an increased tendency to anthropomorphise non-human objects and entities (e.g., pets, products; Epley et al., 2008a,b) and anthropomorphised (vs. non-anthropomorphised) products are more capable of satisfying the social belonging needs of the user (Mourey et al., 2017). Thus, consumers may strengthen their connections to their possessions to cope with their loneliness. Although attaching to possessions in response to loneliness may temporarily reduce loneliness, it may crowd out social connections. Supporting this possibility, a bidirectional relationship between loneliness and materialism over time was observed in a 6-year longitudinal study (Pieters, 2013). Lonely people may also see a brand as a substitute for social connection. It has been found that social exclusion increases preferences for anthropomorphised brands (Chen et al., 2017). Moreover, different types of loneliness have distinct effects on brand preferences. Loneliness stemming from inadequate relationship quality increases brand loyalty, whereas loneliness stemming from inadequate relationship quantity increases brand assortment preference (Jang and Arens, 2023). Finally, consumers may turn to used products as another indirect way to restore feelings of social connection. It has been found that loneliness increases people’s preference for used products because they provide a symbolic connection to previous users (Huang and Fishbach, 2021).

Brand love is conceptualized as a relationship between a consumer and a brand (see Alvarez et al., 2023, for a review of different types of consumer-brand relationships), and is defined as “the degree of passionate emotional attachment a satisfied consumer has for a particular trade name” (Carroll and Ahuvia, 2006, p. 81). Research suggests several antecedents to brand love. For example, products that are more hedonic and brands that afford more self-expressions predict higher levels of brand love (Carroll and Ahuvia, 2006). Moreover, positive brand experience could lead to brand love (de Oliveira Santini et al., 2018) and positive interpersonal experience (e.g., partner quality, social support) in service settings could enhance brand love (Long-Tolbert and Gammoh, 2012). In addition, brand love can also be predicted from brand trust (Albert and Merunka, 2013; Karjaluoto et al., 2016), brand credibility (Bairrada et al., 2018), as well as positive evaluation towards the brand (Arghashi et al., 2021; Han et al., 2019). From the perspective of chronic individual differences among consumers, it has been found that romanticism—a personality trait characterized by being sensitive, chaotic, emotional, and risk taking (Holbrook and Olney, 1995)—is positively associated with brand love (Sarkar et al., 2012). Moreover, materialism—a belief that material possessions reflect success and bring happiness in life (Richins and Dawson, 1992)—is also positively associated with brand love (Ahuvia et al., 2020). Brand love can lead to various positive consequences, contributing to an overall increase in long-term profitability for a brand. For instance, Carroll and Ahuvia (2006) found that brand love is positively associated with brand loyalty and positive word of mouth. In addition, purchase intention is found to be predicted by brand love (Fetscherin, 2014). Furthermore, brand love could also lead to higher willingness to pay a premium price (Thomson et al., 2005).

One factor that could potentially increase brand love is loneliness. As previously reviewed, loneliness would motivate an individual to restore social connection in order to satisfy their need to belong. As previously mentioned, lonely people may view brands as substitutes for social connection. Thus, one perspective is that loneliness would increase emotional attachment to a brand (i.e., brand love) because it serves as a surrogate for social affiliation. Another perspective, on which the present research focuses, is that loneliness would increase emotional attachment to a brand because it serves as a symbolic link connecting people associated with the brand in different roles. These include the CEO and employees of the brand, as well as its users. Thus, a Tesla car owner is indirectly connected to Elon Musk. Likewise, an iPhone user is indirectly connected to staff members at Apple stores. And a Nike wearer is indirectly connected to all other Nike wearers. All of these indirect connections are afforded by the common brand with which people are associated.

According to social identity theory (Tajfel and Turner, 1979), individuals construct their identities partly through the social groups to which they belong. Two members of the same social group may feel that they are tied to each other through a shared social identity, even though there is not any interpersonal relationship between them. Indeed, it has been found that one’s tendency to engage in conversation and sensual interactions (e.g., sharing a bottle of water) with a stranger can be enhanced by having a shared social identity between them (Neville et al., 2022). Moreover, novel faces of the same social group (e.g., students who are affiliated with the same university) are more likely to be recognized (Bernstein et al., 2007) and strangers who belong to the same social group (e.g., people who share the same art preference) are treated more favorably (Tajfel, 1970). Hence, it is possible that loneliness would increase a consumer’s emotional attachment to a brand in order to satisfy their social belongingness need by gaining a greater sense of connection with other people who are associated with the same brand (e.g., other users) and thus belong to the same broad social group, even in the absence of any interpersonal relationship between the consumer and these other people. From my reading of the literature, direct experimental examination of whether loneliness would enhance brand love is lacking. Nevertheless, Loh et al. (2021) reported a positive correlation (*r* = 0.10) between chronic loneliness and degree of emotional attachment to a brand. However, causality inference cannot be drawn from this correlational result. Thus, it remains unknown whether loneliness would cause an increase in brand love.

## 3 Culture and group processes

Social and consumption behaviors, oftentimes, vary as a function of culture (Smith et al., 2013; Shavitt et al., 2020). Culture can be conceptualized in different ways. One way to conceptualize culture is by the values that a group of people share and have internalized. One such cultural orientation that has received the lion’s share of research attention is individualism/collectivism. In a nutshell, people who engage in individualistic cultural contexts tend to define themselves as separate, independent, and autonomous individuals, and prioritize personal goals over collectivistic goals (Markus and Kitayama, 1991; Triandis, 1995). By contrast, people who engage in collectivistic cultural contexts tend to define themselves based on social embeddedness and as fundamentally interdependent with important close others (e.g., family members, close friends, close colleagues), and prioritize collective goals over personal goals (Markus and Kitayama, 1991; Triandis, 1995). Individualistic/Collectivistic cultural orientation can be examined at different levels. At the country level, Western nations (e.g., U. S., Canada, the Netherlands) tend to be relatively individualistic, whereas many non-Western nations (e.g., China, Japan, Mexico, Albania) tend to be relatively collectivistic (Hofstede et al., 2010; Kim, 1994; Markus and Kitayama, 1991; Triandis, 1995). There are also regional variations in individualism/collectivism within a country. For example, within the United States, states in the Deep South (e.g., Louisiana, South Carolina) are relatively collectivistic, whereas states in the Mountain West and Great Plains (e.g., Montana, Oregon) are relatively individualistic (Vandello and Cohen, 1999). In Japan, residents of Hokkaido (the northernmost prefecture) are more individualistic than residents of other regions of the country (Kitayama et al., 2006). Racial/ethnic variations in individualism/collectivism within a country are also evident, paralleling cross-national differences as people tend to retain cultural values of their countries of origin. For example, in the United States, Asian Americans are more collectivistic than European Americans (Park and Kim, 2008; Singelis, 1994). Likewise, European Canadians are more individualistic (or less collectivistic) than East Asian Canadians (Ng et al., 2021). Finally, some researchers have examined individualism/collectivism as individual differences in individualistic/collectivistic cultural orientation (e.g., Holland et al., 2004; Tams, 2008).

Despite the differences in how the self is defined in relation to others between individualistic and collectivistic cultures, social belongingness need certainly applies to all humans (Guisinger and Blatt, 1994; Baumeister and Leary, 1995). Thus, all people exhibit psychological tendencies aiming at satisfying social belongingness need. Yet, group-based psychological processes differ between people engaging in individualistic vs. collectivistic cultural contexts (Yuki and Takemura, 2013). Specifically, individualistic and collectivistic people differ in how they conceptualise ingroups and relate to others who merely belong to the same social category or broad social group (without pre-existing interpersonal relationships). Collectivistic people tend to set a clear boundary between ingroups based on close interpersonal relationships (e.g., family members, close friends) and the outgroup (all others without a close interpersonal relationship). On the other hand, people in individualistic cultures tend to satisfy their social belongingness need from a broader set of ingroups, including not only relational ingroups (i.e., ingroups based on close interpersonal relationships) but also symbolic categorical ingroups (i.e., ingroups based merely on a shared social category or broad social group, not interpersonal relationships). Focusing on symbolic categorical ingroups, Yuki et al. (2005) found that American participants exhibited a higher level of ingroup bias in trust towards strangers of the same town of residence or the same university than did Japanese participants. Moreover, Snibbe et al. (2003) documented that, when evaluating other students who belong to their universities, Japanese (vs. American) students were less likely to exhibit evaluative bias towards them. Finally, Ng et al. (2016) observed that whereas European Canadian participants had a better face memory for novel targets who belonged to the same broad social group (strangers who were affiliated with the same university or belonged to the same personality group), compared with those who belonged to a different broad social group, East Asian Canadians did not exhibit this own-group face recognition bias. The own-group face recognition bias observed in individualistic cultures may reflect an underlying motivation to connect with others affiliated with the same broad social group (Hehman et al., 2010; Wilson et al., 2014). As such, the lack of own-group face recognition bias among people of East Asian background seems to reflect how merely knowing that a stranger belongs to the same board social group would not enhance East Asians’ motivation to connect with the stranger. This is consistent to the exclusive nature of the ingroup in some East Asian cultures documented in early research. For example, Bond (1991) noted that the Chinese tend to “make a critical distinction between established acquaintances and others” (p. 51).

A brand symbolically connects all people affiliated with the same brand (e.g., all Apple users), constituting a broad social group consisting of a large number of people who merely share a common brand affiliation. As previously suggested, loneliness may strengthen people’s emotional attachment to a brand because the brand affords a sense of connection with other people affiliated with the same brand. However, from this perspective, the extent to which loneliness would strengthen brand love should depend on the meaning of shared brand affiliation. Cultural differences in how people conceptualize ingroups and relate to people who merely belong to the same broad social group (Bond, 1991; Yuki and Takemura, 2013) suggest that the effect of loneliness on brand love might differ as a function of culture. In individualistic cultures where people tend to view other people who belong to the same broad social group as ingroup members, even with no preexisting interpersonal relationship, loneliness might indeed increase their emotional attachment to a brand as the brand provides a sense of connection with others affiliated with the same brand. By contrast, in collectivistic cultures where people tend to view all other people with no preexisting interpersonal relationship as the outgroup, emotional attachment to a brand might not be as likely to increase in response to loneliness, despite that the brand provides a sense of connection with other people affiliated with the same brand.

## 4 The present research

The forgoing analyses suggest the following hypotheses:

*H1*: Culture would moderate the effect of loneliness on brand love.

*H1a*: For individualistic participants, loneliness would cause an increase in brand love.

*H1b*: For collectivistic participants, the positive causal effect of loneliness on brand love would be smaller or non-existent.

To examine these hypotheses, two experimental studies with loneliness manipulated were conducted. To ensure that the obtained effect was indeed due to loneliness, rather than the closely related but yet distinct construct—general affect, a confound check measure of general affect was employed, in addition to the manipulation check measure of loneliness. And the hypotheses were tested with general affect used as a covariate. To increase robustness, culture was operationalized differently across the two studies. In Study 1, culture was operationalized as individual differences in individualistic and collectivistic cultural orientations within the same country (United Kingdom). In Study 2, culture was operationalized as racial background within the same country (see Briley et al., 2000; Williams and Aaker, 2002), comparing (collectivistic) East Asian American and (individualistic) White American participants in the United States. Relative to comparing participants from different countries, comparing participants of different racial backgrounds within the same country carries the advantage of minimizing potential confounds that are commonly associated with country (e.g., language, weather, economic condition).

## 5 Study 1

### 5.1 Materials and methods

Two hundred and two Britons (racial background^1^: 164 White, 12 Asian, 13 Black, 8 other/mixed; gender^2^: 78 male, 118 female, 2 non-binary; age^3^: *M* = 39.4), recruited from the Prolific Academic panel, participated in the study for a small monetary compensation. For the brand love measure (see below), two participants did not mention a brand (“none”) or one particular brand (“store brands”), and thus were excluded from data analyses.

After indicating consent, participants first completed the 16-item Cultural Orientation Scale (Triandis and Gelfand, 1998) as a measure of individualistic and collectivistic cultural orientations using a 7-point scale (1 = strongly disagree, 7 = strongly agree). This scale has eight items tapping into facets of individualistic cultural orientation (e.g., “It is important that I do my job better than others”) (α = 0.67) and eight items tapping into facets of collectivistic cultural orientation (e.g., “Parents and children must stay together as much as possible”) (α = 0.79). Following this, loneliness was manipulated using a recall task adapted from Jiao and Wang (2018). Participants were randomly assigned to the lonely condition or the non-lonely condition. In the lonely condition, participants were asked to recall a time when they felt very lonely (e.g., feeling isolated, not having a high sense of intimacy, companionship, friendship, togetherness or feelings of belonging) and describe the experience in as much detail as possible. In the non-lonely condition, participants were asked to recall a time when they felt very connected (e.g., having a high sense of intimacy, companionship, friendship, and feelings of belonging and being loved) and describe the experience in as much detail as possible. Then, participants were asked to indicate the degree to which they felt lonely and disconnected (two items) using a 7-point rating scale (1 = not at all, 7 = very much). An index of loneliness was derived from the mean of these two items (*r*(200) = 0.81), serving as the manipulation check. Then, participants were asked to indicate their general affect using four 7-point semantic differential scales (1 = bad, 7 = good; 1 = unfriendly, 7 = friendly; 1 = unpleasant, 7 = pleasant; 1 = sad, 7 = happy). An index of general affect was derived from the mean of these four items (α = 0.92), serving as a confound check. Finally, participants were asked to indicate a brand that they frequently bought and used, and were satisfied with its products, and then complete the 10-item scale of brand love (Carroll and Ahuvia, 2006) with reference to the brand that they indicated using a 7-point rating scale (1 = strongly disagree, 7 = strongly agree; α = 0.89). Sample items were “I am passionate about XXX”; “I’m very attached to XXX”; and “I have no particular feelings about XXX” (reversed).

### 5.2 Results and discussion

Across 200 participants, 130 unique brands have been mentioned, spanning a range of different product categories, such as apparel and footwear (e.g., Nike, Zara), technology and electronics (e.g., Apple, Sony), personal care and beauty products (e.g., Dove, Nivea), and food and beverages (e.g., Pepsi, Walkers). The 2 most frequently mentioned brands were Nike (*n* = 12, 6%) and Apple (*n* = 10, 5%). All other brands were mentioned by four or fewer participants, with 102 brands (78.5%) mentioned by only one participant. Overall, a highly diverse set of brands was used in the brand love measure across conditions.

Using the index of loneliness, it was confirmed that the manipulation of loneliness was successful. Participants in the lonely condition reported higher levels of loneliness (*M* = 4.16, *SD* = 1.87) than did those in the non-lonely condition (*M* = 2.70, *SD* = 1.62), *t*(198) = 5.86, *p* < 0.001, *d* = 0.83.^4^ Using the index of general affect, it was found that the manipulation of loneliness also induced different levels of general affect. Participants in the lonely condition reported lower levels of positive affect (*M* = 4.25, *SD* = 1.47) than did those in the non-lonely condition (*M* = 5.28, *SD* = 1.47), *t*(198) = −4.94, *p* < 0.001, *d* = 0.70.^5^ Thus, general affect was used as a covariate in the analyses reported below.

**Figure 1:**
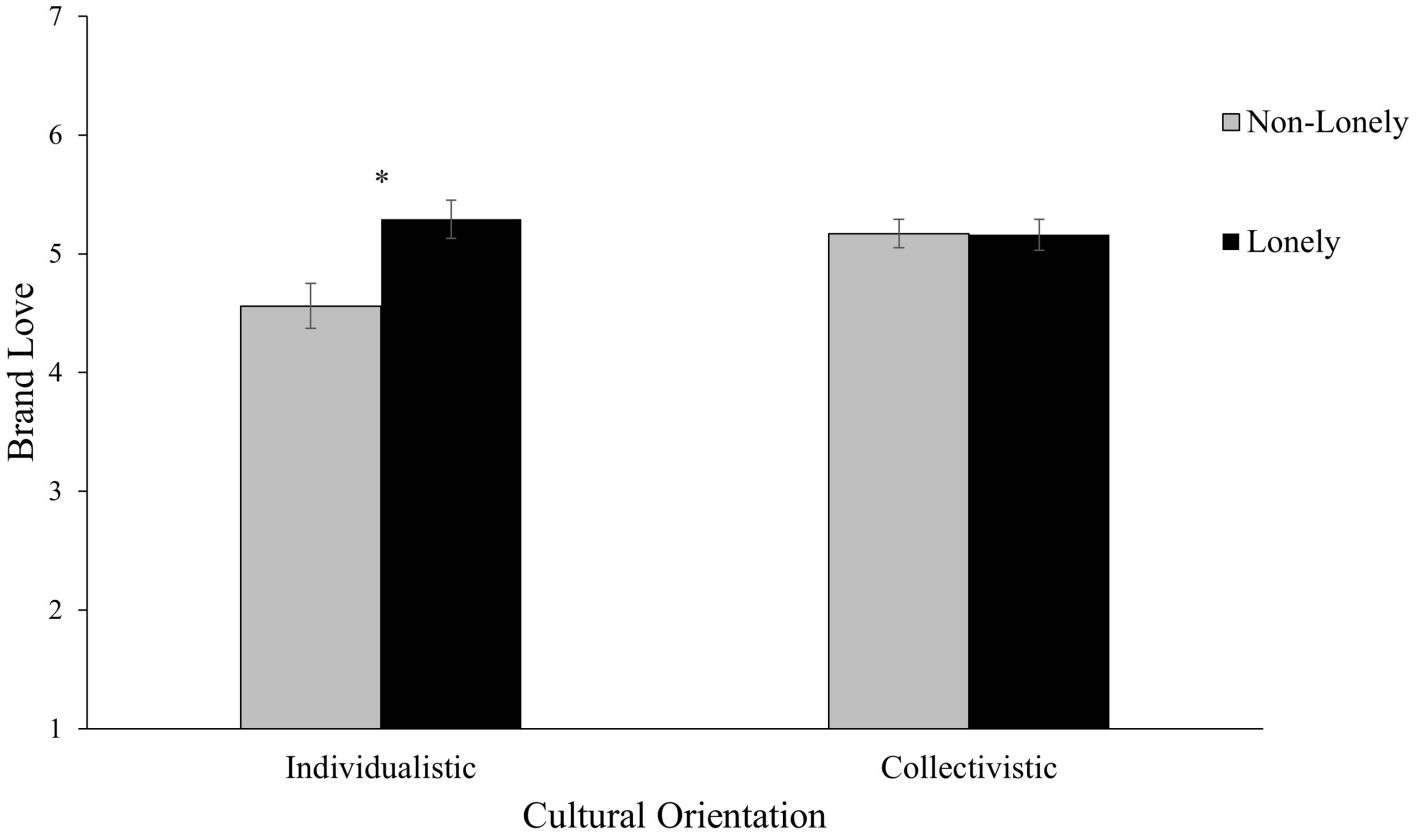
Study 1: Brand love as a function of cultural orientation and loneliness in the United Kingdom. General affect was included as a covariate. Adjusted means are presented. Error bars indicate standard errors. *p < 0.01.

Participants were classified into an individualstic group and a collectivistic group according to their cultural orientation scores. For each participant, if the individualistic cultural orientation score was higher than the collectivistic cultural orientation score, the participant was classified as belonging to the individualstic group. Conversely, if the collectivistic cultural orientation score was higher than the individualistic cultural orientation score, the participant was classified as belonging to the collectivistic group. There were 61 individualistic participants and 128 collectivistic participants (11 participants were neither individualstic nor collectivistic). Then, a 2 Cultural Orientation (individualistic vs. collectivistic) × 2 Loneliness (lonely vs. non-lonely) between-subjects ANCOVA on brand love with general affect as the covariate was conducted to test the hypothesis that culture would moderate the effect of loneliness on brand love. As hypothesized, results revealed an interaction effect between Cultural Orientation and Loneliness, *F*(1, 184) = 6.14, *p* = 0.014, η_p_^2^ = 0.03, supporting H1. Simple main effect analyses indicated that for individualistic participants, those who were in the lonely condition expressed higher levels of brand love (*M*_adj_ = 5.29, *SE* = 0.16) than did those in the non-lonely condition (*M*_adj_ = 4.56, *SE* = 0.19), 95% CI of the difference = [0.249, 1.218], *F*(1, 184) = 8.92, *p* = 0.003, η_p_^2^ = 0.05, consistent with H1a. By contrast, for collectivistic participants, there was no statistically significant difference between brand love of participants in the lonely condition (*M*_adj_ = 5.16, *SE* = 0.13) and those in the non-lonely condition (*M*_adj_ = 5.17, *SE* = 0.12), 95% CI of the difference = [−0.353, 0.341], *F*(1, 184) < 0.01, *p* = 0.972, η_p_^2^ < 0.01, in line with H1b (see Figure 1).^6^

For an alternative analysis, a composite score of relative individualistic cultural orientation was calculated by subtracting the collectivistic cultural orientation score from the individualistic cultural orientation score (Agrawal et al., 2007; Lee et al., 2000). Then, multiple linear regression was used to test the hypothesis that culture would moderate the effect of loneliness on brand love. General affect (grand-mean-centered), relative individualistic cultural orientation (grand-mean-centered), loneliness (0 = non-lonely, 1 = lonely), and the interaction term of relative individualistic cultural orientation and loneliness were used as predictors. Brand love was used as the criterion. Results revealed that the overall regression model was statistically significant, *F*(4, 195) = 5.11, *p* < 0.001, *R*^2^ = 0.10. Importantly, the predicted moderation effect emerged. The interaction term of relative individualistic cultural orientation and loneliness was statistically significant, β = 0.26, *p* = 0.012, *f*^2^ = 0.03, supporting H1. Simple slope analyses revealed that for participants who were high (+1SD) on relative individualistic orientation, those who were lonely (vs. non-lonely) exhibited higher levels of brand love, *b* = 0.58, *p* = 0.003, consistent with H1a. On the other hand, for participants who were low (−1SD) on relative individualistic orientation, the effect of loneliness on brand love was not statistically significant, *b* = −0.11, *p* = 0.582, in line with H1b.^7^

These results indicate that for individualistic (but not collectivistic) consumers, loneliness enhanced their love relationship with a brand. This suggests that brand love may serve as a way to cope with loneliness but this way of coping is present for individualistic consumers only.

## 6 Study 2

### 6.1 Materials and methods

One hundred fifty-three White Americans (72 male, 81 female; *M*_age_ = 45.1) and 115 East Asian Americans (55 male, 58 female, 2 non-binary; *M*_age_ = 35.2), recruited from the Prolific Academic panel, participated in the study for a small monetary compensation.^8^ One East Asian American participant did not pass an attention check question (“please choose the third option from the left”) and thus was excluded from data analyses.

After indicating consent, participants were randomly assigned to the lonely condition or the non-lonely condition (same as Study 1). Following this, participants completed the two manipulation check questions (*r*(267) = 0.88) and the four confound check questions (α = 0.96) (same as Study 1). Finally, participants were asked to complete the 10-item scale of brand love (α = 0.93) (Carroll and Ahuvia, 2006) for a brand that they frequently bought and used, and were satisfied with its products (same as Study 1).

### 6.2 Results and discussion

For the brand love measure, across 267 participants, 153 unique brands were mentioned, spanning a range of different product categories, such as apparel and footwear (e.g., Adidas, Nike), technology and electronics (e.g., Apple, Samsung), personal care and beauty products (e.g., Cetaphil, Dove), and food and beverages (e.g., Hershey’s, Pepsi). The two most frequently mentioned brands were Apple (*n* = 30, 11.2%) and Nike (*n* = 23, 8.6%). All other brands were mentioned by eight or fewer participants, with 119 brands (77.8%) mentioned by only one participant. Overall, a highly diverse set of brands was used in the brand love measure across conditions.

Using the index of loneliness, it was confirmed that the manipulation of loneliness was successful. Participants in the lonely condition reported higher levels of loneliness (*M* = 4.53, *SD* = 1.92) than did those in the non-lonely condition (*M* = 2.52, *SD* = 1.77), *t*(265) = 8.90, *p* < 0.001, *d* = 1.10.^9^ Using the index of general affect, it was found that the manipulation of loneliness also induced different levels of affect. Participants in the lonely condition reported lower levels of positive affect (*M* = 4.16, *SD* = 1.64) than did those in the non-lonely condition (*M* = 5.47, *SD* = 1.33), *t*(258.56) = −7.22, *p* < 0.001, *d* = 0.88.^10^ Thus, general affect was used as a covariate in the analyses reported below.

**Figure 2:**
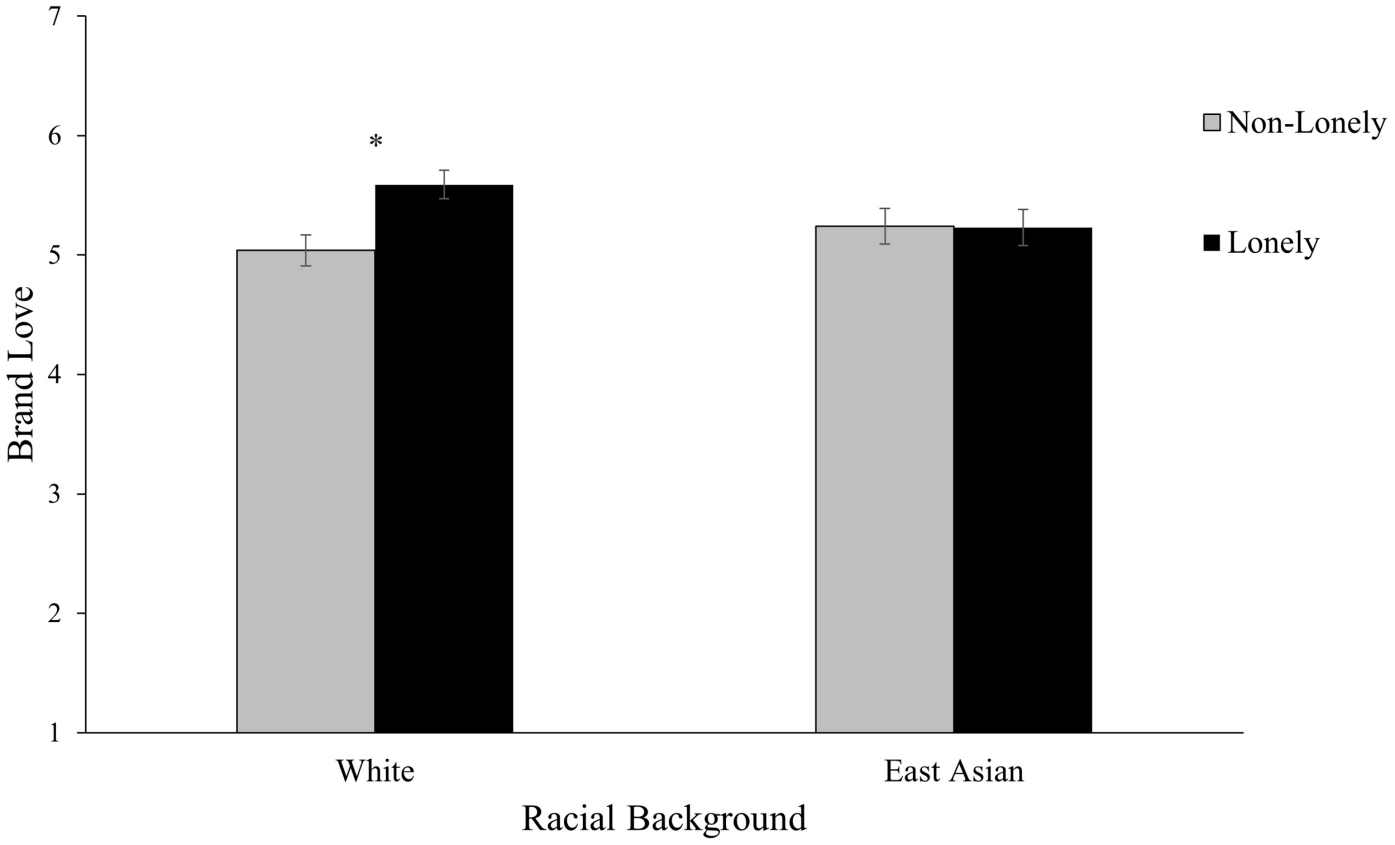
Study 2: Brand love as a function of racial background and loneliness in the United States. General affect was included as a covariate. Adjusted means are presented. Error bars indicate standard errors. *p < 0.01.

A 2 Racial Background (White vs. East Asian) × 2 Loneliness (lonely vs. non-lonely) between-subjects ANCOVA on brand love with general affect as the covariate was conducted to test the hypothesis that culture would moderate the effect of loneliness on brand love. As hypothesized, results revealed an interaction effect between Racial Background and Loneliness, *F*(1, 262) = 4.46, *p* = 0.036, η_p_^2^ = 0.02, supporting H1. Simple main effect analyses indicated that for White American participants, those who were in the lonely condition expressed higher levels of brand love (*M*_adj_ = 5.59, *SE* = 0.12) than did those in the non-lonely condition (*M*_adj_ = 5.04, *SE* = 0.13), 95% CI of the difference = [0.209, 0.904], *F*(1, 262) = 9.96, *p* = 0.002, η_p_^2^ = 0.04, consistent with H1a. For East Asian American participants, on the other hand, there was no statistically significant difference between brand love of participants in the lonely condition (*M*_adj_ = 5.23, *SE* = 0.15) and those in the non-lonely condition (*M*_adj_ = 5.24, *SE* = 0.15), 95% CI of the difference = [−0.419, 0.444], *F*(1, 262) < 0.01, *p* = 0.955, η_p_^2^ < 0.01, in line with H1b (see Figure 2).^11^

Using racial background as the operationalization of culture, these results suggest that for individualistic (White American) consumers, loneliness increased their love towards a brand. For collectivistic (East Asian American) consumers, loneliness did not increase their brand love. Conceptually replicating Study 1, these results provide further evidence that brand love may serve as a way to cope with loneliness that is specific to consumers with an individualistic cultural background.

## 7 General discussion

Experiencing loneliness is a common human experience. When one feels lonely, it is understandable that they would be motivated to restore their social belongingness need to a desired level. This can be achieved by either reestablishing connections with other people directly or restoring a sense of social connection indirectly through other means. Focusing on the indirect route, the purpose of the present research is to investigate whether the feeling of loneliness would enhance people’s emotional connection with a brand. The answer is a conditional yes. Across two studies, I have shown that loneliness can indeed strengthen consumers’ love relationship with a brand. However, this effect is subject to an important boundary condition. Consumers’ cultural background, as operationalized as individual differences in cultural orientation (Study 1) or racial background (Study 2), moderated the causal effect of loneliness on brand love. Specifically, loneliness can cause an increase in brand love for individualistic consumers, but not collectivistic consumers. It is important to note that in both studies, general affect was controlled for, so the effect of loneliness on brand love for individualistic participants demonstrated in this research reflected the unique contribution of loneliness, rather than that mixed with a more general affective state. These results are consistent with current theorizing and empirical findings about how individualistic and collectivistic cultures differ in their conceptualization of ingroup and relate to strangers who belong to the same broad social group (Ng et al., 2016; Yuki and Takemura, 2013). For individualistic consumers who use a brand and are satisfied with its products, all other people who are affiliated with the same brand may be considered as ingroup members. Thus, for individualists, to cope with their loneliness, they may be motivated to increase their sense of social connection, albeit indirectly, through their increased emotional connection with a brand. By contrast, for collectivistic consumers who use a brand and are satisfied with its products, all other people who are affiliated with the same brand may still be considered as outgroup members, as long as they do not have any close interpersonal relationships with these people. Thus, for collectivists, increasing their sense of connection with these people indirectly through emotional connection with a brand is unlikely to be as meaningful. To cope with their loneliness, it may be more meaningful for collectivists to enhance their sense of connection with relational ingroup members, such as family members and close friends, through increasing their emotional attachment to their possessions that provide symbolic connection with these close others. This is consistent to prior research showing how strangers who merely belong to the same board social group are not psychologically treated as “ingroup members” by people who engage in collectivistic cultural contexts (Ng et al., 2016).

The present investigation increases our understanding of the factors that could increase brand love. The positive effect of loneliness on brand love among individualistic consumers documented in the present research has practical implications for marketers. In individualistic cultural contexts, it would be advantageous for marketers to target lonely (vs. non-lonely) consumers as a stronger emotional connection could be formed between these consumers and the brand. This can be achieved by targeting using markers of loneliness, such as being young (Barreto et al., 2021), living alone (Beutel et al., 2017), and being an immigrant (Delaruelle, 2023). The positive effect of loneliness on brand love among individualistic consumers could also be capitalized using promotional materials that include a reminder of how consumers may feel lonely from time to time. Including such a reminder should increase consumers’ love towards the brand, which is known to be associated with a set of desirable consumption outcomes (e.g., brand loyalty, Carroll and Ahuvia, 2006; increased willingness to pay a premium price, Thomson et al., 2005).

The present research has certain limitations that future research could address. First, although the current research includes two operationalizations of culture (individual differences in cultural orientation, racial background), robustness is still limited. Future research could further examine the moderating role of culture in the effect of loneliness on brand love using other operationalizations of culture (e.g., country, region within a country). Second, although loneliness was manipulated in the current research, culture was not. Thus, the results observed in the present studies regarding the moderating role of culture (individualism vs. collectivism) in the effect of loneliness on brand love does not allow causal inference. This is a limitation that future research could address by manipulating both culture and loneliness. Third, the current research focuses on the examination of how loneliness would cause an increase in brand love, and how this effect differs across cultures. As such, whether loneliness-induced increase in brand love is effective in ameliorating some negative consequences of loneliness (e.g., reduced life satisfaction, Schumaker et al., 1993) and how this might differ across cultures remain unexplored. This is another limitation that future research could address. Finally, and more broadly, future research could build on the current work to investigate how culture might moderate the effect of loneliness or social exclusion on some consumption behaviors that foster direct connection with other people. For example, the effect of social exclusion on people’s tendency to buy a product that signals a shared broad social group membership (e.g., a university wristband) before interacting with a new person from the same broad social group (Mead et al., 2011) may be stronger for individualists, compared with collectivists.

## Data Availability

The raw data supporting the conclusions of this article will be made available by the authors, without undue reservation.

## Footnotes

^1^Five participants did not report their racial background.

^2^Four participants did not report their gender.

^3^Four participants did not report their age.

^4^Loneliness condition did not interact with individualistic cultural orientation, *p* = 0.325, or collectivistic cultural orientation, *p* = 0.692, in inducing loneliness.

^5^Loneliness condition did not interact with individualistic cultural orientation, *p* = 0.226, or collectivistic cultural orientation, *p* = 0.360, in inducing general affect.

^6^All significant results remained significant and all non-significant results remained non-significant when general affect was not used as a covariate.

^7^All significant results remained significant and all non-significant results remained non-significant when general affect was not used as a covariate.

^8^This study was open to panel members in the United States who were of “White” or “East Asian” ethnic background (according to a pre-screening questionnaire administered by Prolific Academic). However, on the current survey, some participants (*n* = 33) selected an option other than “White (non-Hispanic)” or “East Asian” (these include those who selected “prefer not to answer”) in response to the racial background question. These participants were excluded and their data were never looked at. Only participants who selected “White (non-Hispanic)” or “East Asian” were included.

^9^Loneliness condition interacted with racial background in inducing loneliness, *p* = 0.012. The manipulation of loneliness was stronger in inducing loneliness for East Asian American participants (lonely condition: *M* = 5.02, *SD* = 1.68; non-lonely condition: *M* = 2.34, *SD* = 1.78; *p* < 0.001) than for White American participants (lonely condition: *M* = 4.18, *SD* = 2.02; non-lonely condition: *M* = 2.65; *SD* = 1.77; *p* < 0.001).

^10^Loneliness condition interacted with racial background in inducing general affect, *p* = 0.001. The manipulation of loneliness was stronger in inducing general affect for East Asian American participants (lonely condition: *M* = 3.53, *SD* = 1.39; non-lonely condition: *M* = 5.53, *SD* = 1.38; *p* < 0.001) than for White American participants (lonely condition: *M* = 4.61, *SD* = 1.67; non-lonely condition: *M* = 5.42; *SD* = 1.29; *p* < 0.001).

^11^All significant results remained significant and all non-significant results remained non-significant when general affect was not used as a covariate.
